# Repellent and Attractant Guidance Cues Initiate Cell Migration by Distinct Rear-Driven and Front-Driven Cytoskeletal Mechanisms

**DOI:** 10.1016/j.cub.2018.02.024

**Published:** 2018-03-19

**Authors:** Louise P. Cramer, Robert R. Kay, Evgeny Zatulovskiy

**Affiliations:** 1 Laboratory of Molecular Cell Biology and Department of Cell and Developmental Biology, Faculty Life Science, UCL, Gower Street, London WC1E 6BT, England, UK; 2 MRC Laboratory of Molecular Biology, Francis Crick Avenue, Cambridge Biomedical Campus, Cambridge CB2 0QH, England, UK

**Keywords:** actin cytoskeleton, myosin II, actin dynamics, myosin dynamics, cell migration, chemotaxis, cell rear retraction, repulsive cell guidance, chemo-repellent, attractive cell guidance

## Abstract

Attractive and repulsive cell guidance is essential for animal life and important in disease. Cell migration toward attractants dominates studies [1, 2, 3, 4, 5, 6, 7, 8], but migration away from repellents is important in biology yet relatively little studied [5, 9, 10]. It is widely held that cells initiate migration by protrusion of their front [11, 12, 13, 14, 15], yet this has not been explicitly tested for cell guidance because cell margin displacement at opposite ends of the cell has not been distinguished for any cue. We argue that protrusion of the front, retraction of the rear, or both together could in principle break cell symmetry and start migration in response to guidance cues [16]. Here, we find in the *Dictyostelium* model [6] that an attractant—cAMP—breaks symmetry by causing protrusion of the front of the cell, whereas its repellent analog—8CPT—breaks symmetry by causing retraction of the rear. Protrusion of the front of these cells in response to cAMP starts with local actin filament assembly, while the delayed retraction of the rear is independent of both myosin II polarization and of motor-based contractility. On the contrary, myosin II accumulates locally in the rear of the cell in response to 8CPT, anticipating retraction and required for it, while local actin assembly is delayed and couples to delayed protrusion at the front. These data reveal an important new concept in the understanding of cell guidance.

## Highlights

• In attractant, cell front protrusion breaks cell symmetry and starts migration
• In repellent, cell rear retraction breaks cell symmetry and starts migration
• Myosin II motor is not required for front-driven migration toward attractant
• Biased myosin II motor contractility drives rear-driven migration away from repellent

## Graphical Abstract

**Figure:**
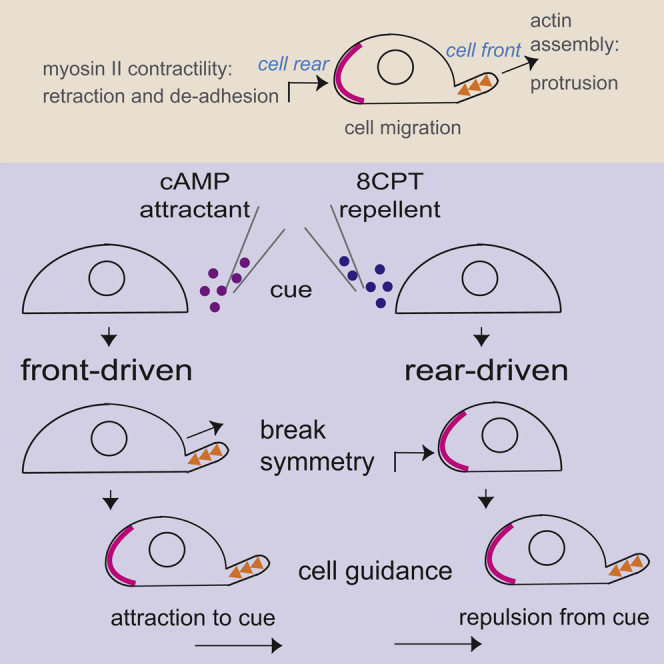

## Teaser

Cell guidance cues are molecules that give “direction-of-travel” instructions—either toward or away from the cue—to migrating cells. Cramer et al. use sufficient temporal resolution to reveal that attractive and repellent cues employ distinct cytoskeleton-force-generating mechanisms to break the symmetry of a cell and start its migration.

## Results and Discussion

To initiate migration, cells must break symmetry (polarize) to establish a protrusive front and a retractive rear. *A priori*, it is not known if attractants and repellents share the same symmetry-breaking mechanism. Therefore, to investigate how cells break symmetry, we used cell margin displacement as a polarity marker because it directly reports cell migration without presupposing any particular mechanism. In principle, either stable, outward movement of the cell front (initial front protrusion) or stable, inward movement of the cell rear (initial rear retraction), or both together, could break cell symmetry and start migration [16]. These alternative mechanisms have very different implications for our understanding of how cells steer in chemotactic gradients and communicate between their front and back.

**Figure 1:**
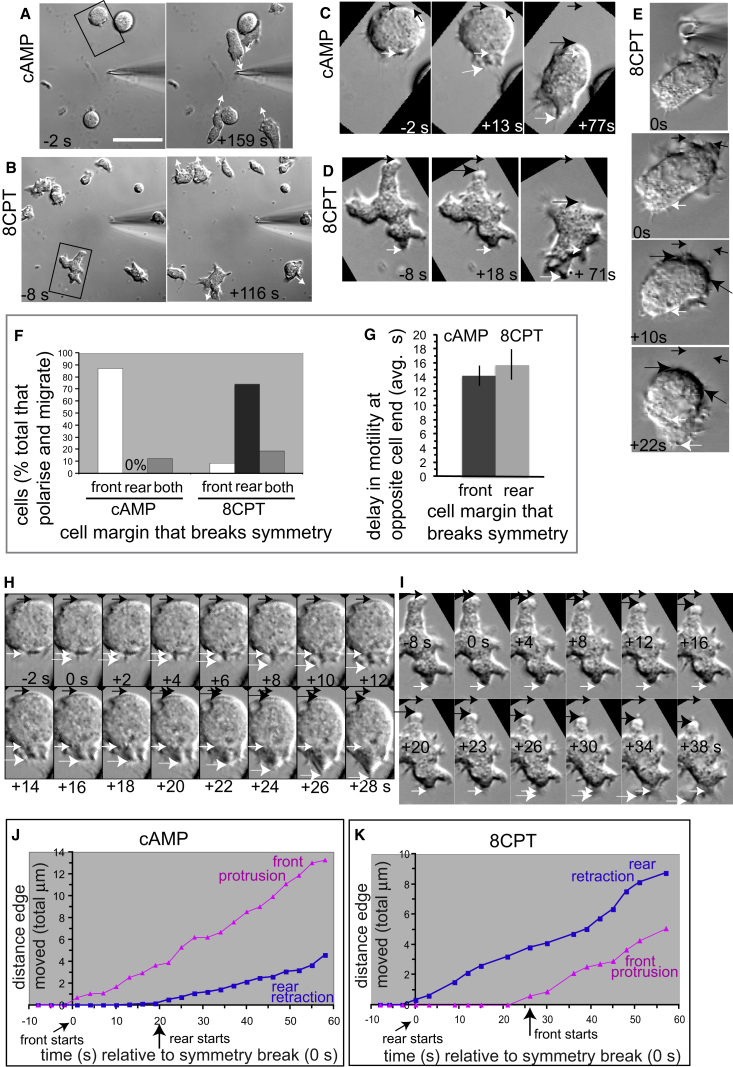
A Repellent and an Attractant Initiate Cell Movement at Opposite Cell Margins cAMP is an attractant for *Dictyostelium* cells, and its analog, 8CPT, is a repellent. (A and B) Images from a time-lapse sequence showing cells moving toward a source of cAMP (A) and away from a source of 8CPT (B). Arrows indicate direction of cell migration. Time is relative to the start (0 s) of polarization for the cells in the boxes. (C–E) Boxed cells (A and B) are rotated in (C) and (D). Short and longer white arrows indicate initial and new position of the cell front, respectively. Short and longer or larger black arrows indicate initial and new position of the cell rear, respectively. Time is relative to the start (0 s) of the break in cell symmetry. (C) Time-lapse images for the cell in the box in (A) showing that symmetry is broken by protrusion of the front of the cell in response to cAMP. (D and E) Time-lapse images for the cell in the box in (B) (D) and for a cell from another sequence that has distinct initial non-polarized shape (E), both showing that symmetry is broken by retraction of the rear of the cell in response to 8CPT. (F) Comparing types of cell margin displacements that break symmetry in cAMP or 8CPT. Plot is proportion of all polarizing cells (n = 24 cells [cAMP] and n = 35 cells [8CPT] from 10–13 experiments per cue). Front, front protrusion; rear, rear retraction; both, front protrusion and rear retraction start together. (G) Delay between initial front protrusion and initial rear retraction for cells in (F) that break symmetry with front protrusion in cAMP (n = 21 cells) and with rear retraction in 8CPT (n = 26 cells). Each value is the mean ± SEM. (H and I) Earliest visual steps during cell polarization from the sequences in (C) and (D) showing that cell margin displacement at the opposite cell end is delayed throughout the break in symmetry in cAMP (H (0–18 s) or in 8CPT (I) (0–23 s). Arrows and time are as in (C–E). (J and K) Time-distance plots of paired cell front and rear margins showing distinct temporal order of their displacement in cAMP (J) compared with 8CPT (K) for the cells in (C) and (D), respectively. AX2 cells were used throughout. Bar (A): 28 μm (A), 30 μm (B), 10 μm (C and H), and 11 μm (D, E, and I). See also Figure S1 and Table S1 and Movies S1 and S2.

Polarized cytoskeletal forces drive cell polarization. For almost all cell types, actin filament assembly or bleb formation in the front of the cell drive protrusion, but in our conditions, blebs were rare and are not studied, while myosin II motor-based and other distinct types of contraction can power retraction of the rear, depending on cellular context [11, 12, 13, 14, 15, 17, 18, 19]. In the wild, *Dictyostelium discoideum* amoebae are guided by both chemo-attractants and chemo-repellents [20, 21]. Here, we use the AX2 strain as “wild-type” cells, cyclic AMP (cAMP), a natural attractant (Figure 1A), and 8-(p-chlorophenylthio)-cAMP (8CPT-cAMP), an analog of cAMP that repels these cells, hereinafter referred to as 8CPT (Figure 1B) [22]. Strong gradients of each cue are used in all experiments.

**Figure 2:**
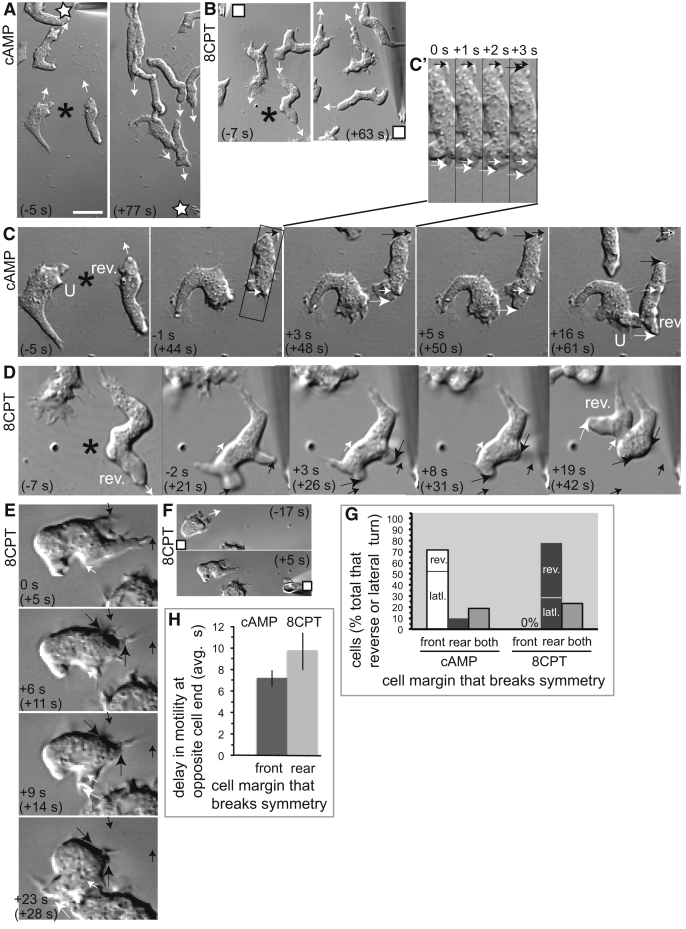
The Repellent 8CPT Initiates Cell Repolarization at the Opposite End of the Cell to the Attractant cAMP in Turning AX2 Cells (A and B) Images from a time-lapse showing that cells turn toward repositioned cAMP (A, star) and away from repositioned 8CPT (B, square). Arrows indicate direction of migration. Time is relative to the start (0 s) of repositioning the guidance cue. (C–F) Arrows indicate as in Figures 1C–1E. Time without brackets is relative to the start (0 s) of cell repolarization. Time within brackets is relative to the start (0 s) of repositioning the guidance cue. U, U-turn (C); rev, reverse cell turn (C and D). (C) Time-lapse images for the two cells indicated with an asterisk in (A). The cell in the box (C and inset) shows that front protrusion leads repolarization toward cAMP. (D–F) Time-lapse images for the cell at the asterisk in (B) (D) and for a cell from another sequence (E and F) that has distinct initial shape, both showing that rear retraction leads repolarization away from 8CPT. Squares (F) indicate repositioning of 8CPT for the cell in (E). (G) Comparing types of cell margin displacement that repolarize cells during reverse and lateral turns in cAMP or in 8CPT. Plot is proportion of all repolarizing cells (n = 28 cells [cAMP] and n = 36 cells [8CPT] from 13–18 reorientation experiments per cue). Abbreviations are as in Figure 1F. Additional cells performed U-turns, and these do not repolarize to turn. (H) Delay between initial front protrusion and initial rear retraction for the cells in (G) that start cell repolarization with front protrusion in cAMP and with rear retraction in 8CPT. Each value is the mean ± SEM. Bar (A): 16.7 μm (A and B), 10 μm (C–E), and 6.7 μm (C inset). See also Table S2.

To begin to identify which cytoskeletal-based forces break cell symmetry and start cell migration, we determined the timing of the initial, stable front protrusion and rear retraction (Figures 1 and 2). The temporal resolution was 1 s, which readily allows these events to be distinguished. For clarity, whichever cell margin displaces first is defined as the “start of migration” (for reference, 0 s in Figures 1H and 1I), while the start of whole-cell translocation is when both initial front protrusion and initial rear retraction have occurred (for reference, from 20 s in Figure 1H and from 26 s in Figure 1I).

### Breaking of Cell Symmetry in Response to cAMP Attractant and 8CPT Repellent

*Dictyostelium*, like many other cells, can migrate randomly in the absence of cell guidance cues. We therefore used a cooling-rewarming protocol that causes most cells to lose polarity, enabling us to capture their repolarization in response to guidance cues after they had been warmed up [23]. We validated (Figures S1A–S1F) that this method faithfully recapitulates reported behaviors during polarization of amoeboid cells [1, 24] and other cell types [25, 26, 27] that have not been priorly cooled and rewarmed. In particular, we confirmed [1, 24, 25, 26, 27] that the shape of non-polarized cells can vary (Figures S1A–S1C) and that unstable protrusions and retractions of the cell periphery occur prior to polarization but do not break symmetry (Figures S1D and S1E). Subsequent to this behavior, polarized displacement of the cell margin breaks symmetry and starts cell migration (Figures S1D and S1E). We tracked and quantified the behavior of 87 cells in response to cAMP and 141 cells in response to 8CPT during the first 200–300 s of their encounter with the guidance cue (Table S1). Of the cells that were non-polarized at the start of filming, about one-third polarized and migrated in the expected direction during the encounter (Table S1). Most of the remaining cells did not polarize during the encounter period (Table S1), though a few moved the wrong way and were disregarded from further analysis (Table S1).

In response to cAMP, cells formed a stable protrusion toward the source of the attractant to break symmetry and start migration (Figures 1C [white, longer arrow] and 1F and Movie S1). Throughout symmetry breaking (Figures 1H [0 s to +18 s; white, longer arrow]), there was little or no change in position of the presumptive cell rear (Figures 1H [compare white and black arrows] and 1J [compare traces] and Movie S1). This reveals that migration was initiated by protrusion of the front of the cell in response to this attractant. Retraction of the rear of the cell was delayed (Figures 1C, 1H [black, longer arrow], and 1J and Movie S1) by about 14 s on average (Figure 1G), and then, the whole cell moved toward the cAMP (Figure 1C [+77 s]).

Conversely, in response to 8CPT, cells broke symmetry and started migration by retracting part of the cell closest to the repellent (the presumptive cell rear) (Figures 1D, 1E [black, longer arrow], and 1F and Movie S2). This occurred similarly for cells of initially flatter (Figure 1D) or rounder (Figure 1E) shape. Throughout the breaking of symmetry (Figures 1I [0 s to +23 s; black, longer arrow]), there was little or no movement of the presumptive cell front (Figures 1I [compare white and black arrows] and 1K [compare traces] and Movie S2). This shows that repulsive migration was initiated by retraction of the cell rear. Protrusion of the opposite end of the cell (Figures 1D, 1E, 1I [white, longer arrow], and 1K) was delayed by about 16 s on average (Figure 1G), after which the whole cell moved away from the source of 8CPT (Figures 1D [+71 s] and 1E [+22 s]).

Once the whole cell had started moving, we could not detect delays between protrusion of the front and retraction of the rear on the same time and imaging scales (Figures S1G and S1H). This is comparable to other front-rear analysis during cell migration [26, 28, 29]. Also, during whole-cell movement, the speeds of front protrusion and rear retraction were essentially the same, with a ratio of around 1:0 (Figure S1I). Thus, by these measures, the protrusion and retraction delays that occur as cells break symmetry are specific to the initiation of migration itself and not to any general difference between the front and rear of the cell.

### Cell Turning

In a distinct experimental approach—and one without cell cooling-rewarming—we studied different types of turns (U, reverse, and lateral) produced when migrating cells are forced to alter direction by changing the position of the chemotactic gradient (Figure 2) [30, 31, 32]. In a U-turn, cells steer around from their front (observable in left-hand cell, Figure 2C), but do not repolarize because they keep their original cell front and rear. Alternatively, just after the gradient is moved, cells stop and typically produce transient, de-localized protrusions and retractions [30] (observable in right-hand cell, Figure 2C). Cells then repolarize by forming a new front and new rear either lateral to the original direction (lateral turn) or at roughly 180° (reverse turn).

Cells turn toward repositioned cAMP (Figure 2A) and away from repositioned 8CPT (Figure 2B). We tracked 86 cells responding to cAMP and 48 cells responding to 8CPT (Table S2), of which 95% and 85%, respectively, performed either U-turns, lateral turns, or reverse turns (quantified in Table S2). Many cells performed U-turns toward re-positioned cAMP (Table S2), similar to other reports [30, 31, 32]. Of note, hardly any cells performed U-turns away from the repositioned 8CPT repellent (Table S2), which may reflect a difference in underlying mechanism.

To turn laterally or reverse toward repositioned cAMP, cells formed a new protrusion (new front) to break symmetry and start migration (Figures 2C [and inset; long white arrows], 2G, and 2H). In contrast, to reverse or laterally turn away from repositioned 8CPT, cells retracted a new rear to break symmetry and start migration (Figures 2D, 2E [long black arrows], 2G, and 2H). This occurred similarly for migrating cells of initially different shape (compare Figures 2D and 2E).

Hence, our findings on cell polarization are similar in two separate experimental systems in *Dictyostelium* cells (Figures 1 and 2). That retraction breaks symmetry predicts that contractile force at the rear, rather than actin assembly at the front, initiates migration of these cells away from the repellent, 8CPT.

### Actin Assembly: Spatial Polarization at the Front

**Figure 3:**
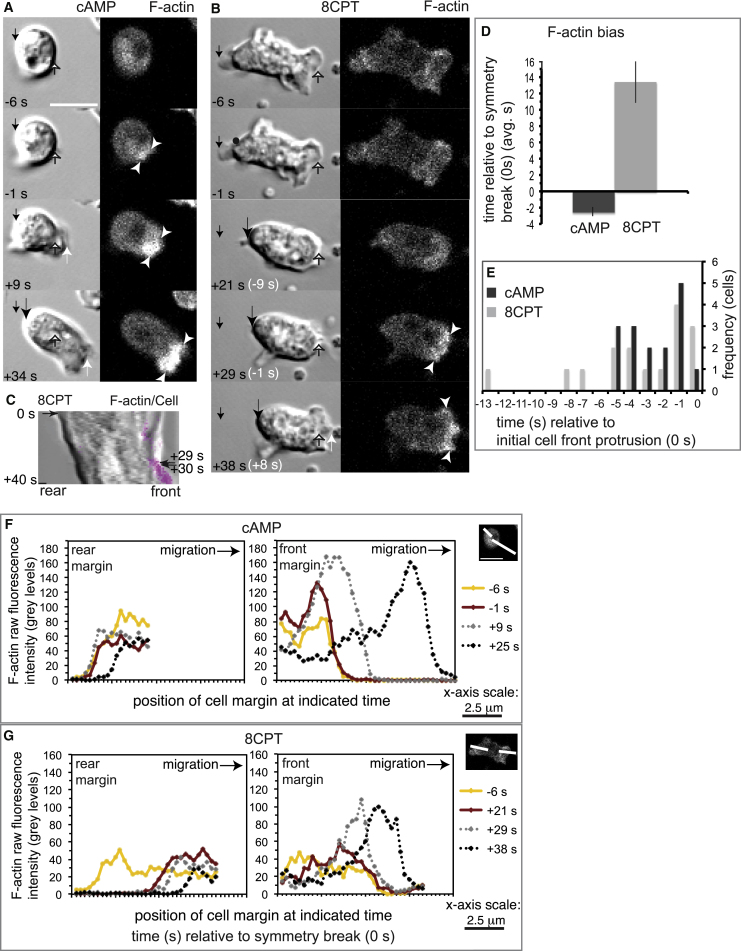
Actin Filaments Polarize to the Front of AX2 Cells in Response to 8CPT Repellent after Symmetry Has Been Broken at the Rear (A and B) Paired cell and actin filament fluorescence images from a time-lapse showing that actin filaments polarize at the cell front 1 s prior to the breaking of cell symmetry in response to cAMP (A, −1 s) and by 29 s after the breaking of cell symmetry in response to 8CPT (B, +29 s). Arrows indicate as in Figures 1C–1E. Time (s) without brackets (A and B) is relative to the break in cell symmetry (0 s). Time (s) within brackets (B) is relative to the initial front protrusion (0 s). (C) Kymograph (time-distance plot) for the cell in (B) showing actin filaments (pink) polarize at the front ∼27–29 s after initial rear retraction starts and ∼1–3 s before front protrusion starts (+30 s) in 8CPT. Note that in 8CPT, actin starts to visibly accumulate at the front toward the end of initial retraction. Time is relative to the break in cell symmetry (0 s). Shown is an overlay of paired actin fluorescence and cell kymograph images. Position of the kymograph is approximately between the dot and white arrow in (B, −1 s). Weaker actin filament fluorescence detectable in (B) is not visible in the kymograph. (D) Timing of visible actin filament spatial bias relative to the start of polarization of the same cell in cAMP or 8CPT gradients. Each value is the mean ± SEM; n = 16 cells [cAMP] and n = 16 cells [8CPT] from 10 experiments per cue; same source films as Figure 1. (E) Comparison of the timing of visible actin filament polarization relative to when the front starts protruding for the cells in (D). Mean ± SEM for cAMP is −2.6 ± 0.4 s and for 8CPT is −3.4 ± 0.9 s. (F and G) Line scans of F-actin fluorescence for paired rear and front zones showing distinct kinetics of actin filament bias in cAMP (F) compared with 8CPT (G) for the polarizing cells in (A) and (B), respectively. Actin increases before the symmetry break in cAMP but afterward in 8CPT. Scan positions are indicated in the insets. Raw fluorescence intensities are plotted. Time is relative to the break in cell symmetry (0 s). Bar (A): 10 μm (A and B) and 8.5 μm (C). See also Movies S3 and S4.

**Figure 4:**
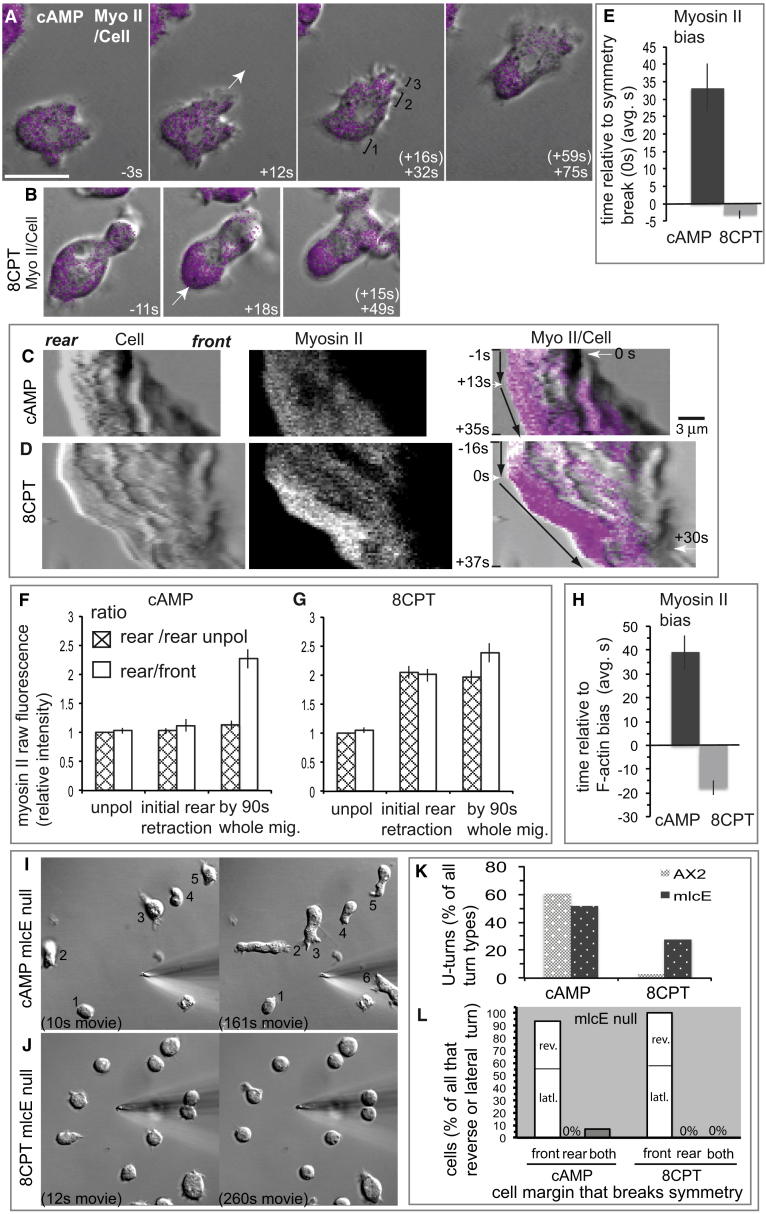
Myosin II Plays a Distinctly Different Role in Cells Responding to 8CPT Repellent Compared to cAMP Attractant (A and B) Overlay of paired images from a time-lapse of an AX2 cell (gray) showing that myosin II (pink) bias develops after polarization during whole-cell translocation in cAMP attractant (A) but during the symmetry break in 8CPT repellent (B). Arrows indicate the symmetry break. Time without brackets is relative to the start of polarization and within brackets to the start of whole movement. In 8CPT (B), encroachment of cell bulk from behind is visible during polarization (also visible in Figure 3B). In cAMP, myosin II bias begins at 21 ± 7 s of whole-cell translocation (mean ± SEM for the cells in cAMP in E). Numbered brackets (A) relate to (F) and (G). (C and D) Kymograph (distance-time plot) of a live AX2 cell showing that myosin II does not polarize during initial cell rear retraction in cAMP (C) but polarizes within the cell rear 2–4 s before its initial retraction in 8CPT (D). Shown are paired kymographs of the cell, myosin II fluorescence, and cell-fluorescence overlay. Time is relative to the break in cell symmetry (0 s). (E) Comparing timing of visible myosin II bias relative to the symmetry break for that cell in AX2 cells in cAMP or 8CPT. Each value is mean ± SEM; n = 15–25 cell rear zones in each cue in 14 (cAMP) or 15 (8CPT) cells from 9–10 experiments per cue; same source films as Figure 1. (F and G) Relative myosin II fluorescence intensity within the cell rear during its initial retraction and during whole-cell translocation (“whole mig”) compared to before polarization (“unpol”) for the same cell and comparing the rear to the front in the same cell in cAMP (F) or 8CPT (G). Each value is the mean ± SEM of individual ratios; n = 19–38 paired rear-rear or paired rear-front zones in 14–15 cells per guidance cue. Brackets (A) refer to the approximate location of the measurement of fluorescence within the rear (bracket 1) and front (bracket 2). Bracket 3 refers to the extreme tip (∼1 μm) of the front and is excluded from measurements (see STAR Methods). (H) Comparison of the timing of myosin II and actin filament polarization during the breaking of symmetry in the same AX2 cell. Each value is mean ± SEM; n = 20–24 paired rear-front zones in 13–14 cells per guidance cue). (I and J) Images from a time-lapse of *mlcE* null cells showing that cells initiate polarization and move toward cAMP (I) and fail to initiate polarization in 8CPT (J) when comparing the first ∼5 min of the guidance cue. Time refers to total elapsed time of the movie. n = 3 (cAMP) and 5 (8CPT) experiments. (K) Comparison of AX2 or *mlcE* null cells that U-turn in response to cAMP or 8CPT in 31–71 cells (13–28 reorientation experiments per condition) as a proportion of total types of turns (U-, reverse, and lateral) for that condition. AX2 data are from Table S2. (L) Type of cell margin displacement that starts reverse and lateral turns in cAMP or 8CPT for the *mlcE* cells in (K) (15–26 cells per cell guidance cue). Bar (A): 10 μm (A and B) and 30 μm (I and J); Bar (C): 3 μm (C and D). See also Figure S2.

To test this idea directly, we determined the dynamics of actin filaments (Figure 3) and myosin II (Figures 4A–4H) in live cells.

In response to cAMP attractant, actin filaments increased within a discrete zone on the side of the cell-facing attractant—the prospective front. This occurred a few seconds prior to the breaking of symmetry (Figures 3A [−1 s] and 3D). Correlated with further filament increase, this actin-rich zone then protruded to break symmetry and start cell migration (Figures 3A [+9 s, +34 s], and 3D–3F and Movie S3). This response was specific to the front, as net increases in actin filaments were not detected elsewhere in the cell (Figures 3A [compare panels] and 3F [compare traces]). These data show that in response to cAMP and in line with other work on actin [33] and other relevant molecules [34, 35], migration starts with an increase in actin assembly at the prospective front of the cell.

In contrast, in response to 8CPT, actin filaments did not increase in the prospective cell front until an average 13 s after retraction of the rear had already broken symmetry (Figures 3B–3D and 3G and Movie S4). This delayed F-actin polarization became evident during the later phases of rear retraction (Figures 3B, 3C [+29 s] and 3G [compare traces] and Movie S4). However, similar to the cAMP case, actin filaments increased at the cell front a few seconds before the start of protrusion (Figures 3B, 3C [compare +29 and +30 s], and 3E and Movie S4). We conclude that migration away from the repellent is not initiated by a spatial bias in actin filament assembly, which instead is linked to the delayed protrusion of the front of the cell.

### Myosin II: Acquisition of Spatial Bias at the Rear

Myosin II is typically diffusely distributed in polarized, amoeboid cells migrating on a two-dimensional surface with a bias toward the rear, whether they are responding to cAMP or 8CPT [22] (Figure S2A–S2C). However, we discovered differences in timing and manner of myosin localization during the initial response of cells to these signals (Figures 4A–4H).

Myosin II only became biased toward the rear of cells exposed to cAMP about an average 33 s after they broke symmetry by extending their front (Figure 4A [+75 s] and 4E). Typically, bias was not detected as the rear started to retract (Figures 4C and 4F) but developed later during whole-cell translocation (Figures 4A and 4F), increasing 2-fold on average (Figure 4F). This bias toward the rear is primarily due to a global decrease in the front (Figures 4A [compare panels] and 4F [compare ratios]). Overall, these data suggest that the spatial bias in myosin II in response to cAMP is a global polarization event linked to early cell translocation.

In contrast, myosin II bias at the rear was linked to the symmetry break (Figures 4B, 4D, 4E, and 4G) and polarized on the side of the cell facing 8CPT a few seconds before it first retracted (Figures 4D and 4E). Myosin II fluorescence increased 2-fold on average (Figure 4G) primarily due to a direct increase within the rear (Figure 4G [compare ratios]), suggesting that the bias in myosin II distribution produced in response to 8CPT is a local polarization event—contrary to the cAMP case. We do not yet know how myosin II polarizes, and several mechanisms are plausible. Whatever the mode, presumably, the relatively lower abundance of F-actin within the rear (evident in Figure 3B) is a sufficient substrate for myosin II to generate force.

### Temporal Order of Actin and Myosin II Polarization

These data must mean that as cells start migrating, F-actin polarizes before myosin II in response to cAMP—as previously reported [33]—and that myosin II polarizes before F-actin in response to 8CPT. This is precisely what we observed in measurements of the timing of actin and myosin II polarization directly within the same cell (quantified in Figure 4H).

### Importance of Myosin II-Motor Based Contractility

As a final test of mechanism, we assessed *Dictyostelium* cells null for myosin II essential light chain (*mlcE*), which form myosin II filaments but have little or no motor-based contractility [36]. The importance of myosin II in cell motility depends on the context [18], but it is agreed that myosin II contractility is not required for *Dictyostelium* polarization, migration, and turning in response to cAMP when the cells move on a two-dimensional surface [33, 37, 38, 39, 40, 41, 42]. The same is true for *mlcE* null cells polarizing in response to this chemo-attractant cue (Figures 4I, 4K, and 4L). And further, as with wild-type cells, protrusion of the front initiates migration (Figure 4L). Our observation that a myosin II spatial bias is not linked to the initial retraction of the rear of cells moving toward cAMP (Figures 4A, 4C, 4E, and 4F) explains why myosin II null cells can polarize toward this guidance cue. We and other groups have identified other modes of contractility that are independent of myosin II motor [18, 42, 43, 44, 45, 46] and suspect that at least some of these must retract the cell rear—at least initially when responding to cAMP. However, during whole-cell translocation, myosin II-motor-based contractility contributes to the speed of rear retraction [37, 38, 39, 41, 42]—as we find for both guidance cues.

Conversely, most *mlcE* null cells failed to polarize and migrate away from 8CPT within the standard 300-s analysis period (Figure 4J): only 6.2% (5/81) did so, which is 5-fold less than wild-type. More cells polarized if left for around 30 min longer in a turning assay. These cells could turn but use a different mechanism than wild-type cells. Retraction of the rear did not break symmetry (Figure 4L) unlike wild-type (Figures 1, 2, 3, 4B, and 4D and Movies S2 and S4), but instead, the cells either polarized by protrusion at the front (Figure 4L) or performed U-turns, indicating steering from the front (Figure 4K). Neither mode was typically observed with wild-type in response to 8CPT (Figures 1F and 2G and Tables S1 and S2). These results strongly suggest that myosin II motor activity is important for symmetry breaking when it starts with rear retraction in these cells.

By studying *Dictyostelium* cells at sufficient temporal resolution, we find explicit evidence that migration toward cAMP fits the widely reported model [11, 12, 13, 14, 15] where actin filament assembly at the front drives protrusion, breaking symmetry and initiating migration. On the contrary, to move away from 8CPT, cells break symmetry by contracting at the rear using myosin II motor activity. In both cases, cell symmetry is broken by a local cytoskeletal response to the guidance cue, but this starts at opposite ends of the cell with distinct cytoskeleton protein activities (depicted in the Figures S2D and S2E). An important outcome of our work is that breaking symmetry from the cell rear does not fit any of the popular theoretical models for amoeboid movement or steering in response to the guidance cue [47].

Myosin II contractility is sufficient for some types of cell migration [48], and both constitutive locomotion [27, 49] and the motility of cell fragments [26] start with rear retraction, hinting that cell symmetry may regularly be broken by contraction of the rear of the cell. We predict [16] that different types of repellents or other conditions that repel cells will turn out to employ this rear-driven mode of cell polarization. Clearly, this needs direct testing for different repellents, conditions, and cells.

Overall, our work reveals that both “front-driven” and “rear-driven” modes of starting migration in response to guidance cues must now be considered. We envisage that these two distinct mechanisms provide an effective means for cells to navigate complex tissue environments and that their recognition will lead to the discovery of important, and as yet unrealized, pathways for the early steps of cell guidance.

## STAR★Methods

### Key Resources Table

**Table:** 

REAGENT or RESOURCE	SOURCE	IDENTIFIER
**Chemicals, Peptides, and Recombinant Proteins**		

cyclic-AMP (cAMP)	Sigma-Aldrich	A9501; CAS: 60-92-4
8-(p-Chlorophenylthio)-cyclic-AMP (8CPT)	BIOLOG	C 010; CAS: 93882-12-3

**Experimental Models: Organisms/Strains**		

*Dictyostelium discoideum* axenic strain AX2 (Kay laboratory strain)	Laboratory of Rob Kay	dictyBase: DBS0235521
*Dictyostelium discoideum* myosin II essential light chain null strain (*mlcE* null)	Dicty Stock Center	dictyBase: DBS0236566

**Recombinant DNA**		

GFP-ABP-120 plasmid	[50]	dictyBase plasmid ID: 472; pDXA-GFPABD120
mRFPmars-ABP-120 plasmid	[51]	dictyBase plasmid ID: 472; mars-ABD120
GFP-myosin II (*mhcA*)	[52]	N/A

**Software and Algorithms**		

MetaMorph	https://www.moleculardevices.com/systems/metamorph-research-imaging/metamorph-microscopy-automation-and-image-analysis-software	RRID: SCR_002368

### Contact for Reagent and Resource Sharing

Further information and requests for resources and reagents should be directed to and will be fulfilled by the Lead Contact, Louise P. Cramer (l.cramer@ucl.ac.uk).

### Experimental Model and Subject Details

#### Dictyostelium Strains

*Dictyostelium discoideum* axenic strain AX2 (Kay laboratory strain; DBS0235521 at http://dictybase.org) used as ‘wild-type’, and myosin II essential light chain null strain (*mlcE*^*−*^, DBS0236566) were used in the experiments. For F-actin visualization, *Dictyostelium* cells were transformed with an F-actin reporter construct consisting of GFP [50] or RFP [51] fused to the F-actin binding domain of *Dictyostelium* protein ABP-120, and for myosin II visualization transformed with the myosin II (*mhcA*) – GFP fusion construct [52].

### Method Details

#### Cell Growth and Developmental Stage

*Dictyostelium* cells were grown on tissue culture plates in axenic medium (HL5 plus glucose medium (Formedium), 200 μg/ml Dihydrostreptomycin) at 22°C [19, 23, 53]. For all experiments, developmental stage was carefully controlled: cells were developed to an aggregation-competent state by first harvesting vegetative amoebae from axenic media and washing them three times in KK_2_ buffer (16.5 mM KH_2_PO_4_, 3.9 mM K_2_HPO_4_, 2 mM MgSO_4_, 0.1 mM CaCl_2_, pH 6.1). After washing, cells were counted and resuspended in KK_2_ buffer at 2 × 10^7^ cells/ml. They were then shaken at 180 rpm at 22°C for one hour (for starvation) before pulsing with 90 nM cAMP (cyclic-AMP, final concentration) every six minutes for 4.5 hours, using a peristaltic pump (Watson Marlow 505D). This ensures that the cAMP receptor and other genes are expressed properly.

#### Treatment of Live Cells with Cell Guidance Cue

All experiments were done with a gradient of cell guidance cue. Aggregation-competent cells were washed in KK_2_ buffer prior to stimulation. Cells were then stimulated directly on the microscope with guidance cue flowing from a glass micropipette (Femptotips II, Eppendorf, Germany) [19, 23] filled with either 2 μM solution (in KK_2_) of chemo-attractant cAMP (cyclic-AMP, Sigma Aldrich), or, as previously reported with, 10 mM [22] of chemo-repellent 8-(*p*-Chlorophenylthio)-cyclic-AMP (BioLog), referred to here as 8CPT. The glass micropipette was positioned using a micromanipulator (Eppendorf 5171, Germany). Diffusion from the micropipette created a steep gradient of guidance cues. In these cells it is thought (and known for cAMP) that 8CPT binds the cAMP receptor, cAR1 [22]. In these cells, cAMP works through G-alpha2; and as far as it has been investigated, 8CPT - through G-alpha1 [22].

#### Breaking of Cell Symmetry Assay

We used live cells in all experiments. This assay reports the break in cell symmetry (also defined as initiation of cell migration) and consequent migration afterward. Aggregation competent *Dictyostelium* cells were prepared (as above), washed in KK_2_ buffer and settled at room temperature in KK_2_ buffer on two-well Lab-Tek chambered microscopy coverslips (Nalge Nunc International, USA). After settling, cells on coverslips were pre-cooled on ice for 10 minutes, which causes cessation of any random cell migration and induces loss of cell polarity [23]. Then immediately, cells were moved to a microscope at room temperature for filming and stimulated with cell guidance cue (as described above) at room temperature. Once on the microscope, encounter with cell guidance cue flowing from the micropipette as cells warmed-up induced the breaking of cell symmetry and initiation of cell migration either toward cAMP or away from 8CPT, which could be captured on film [23]. Cells were filmed typically for 200 s-300 s and occasionally for 100-200 s from the point of the first stable encounter with cell guidance cue. Practically, the time it took to reach the first stable encounter with guidance cue is the time it took to move cells to the microscope, find the micropipette in the field of view and start filming; this was typically 1-2 minutes, but occasionally was 5-7 minutes. Images were collected every 1 s with dual fluorescence and DIC time-lapse microscopy using a Zeiss 710 laser scanning confocal microscope and a 63 × oil-immersion objective (Zeiss, Germany).

##### Cell Behaviors Observed in the Assay

In this assay, for both cell guidance cues, we observed: cells that polarized, cells that did not polarize, cells that were already migrating at the start of filming, and rarely, cells that moved the wrong way. We fully characterized and quantified these behaviors in 87-141 individual cells (Table S1). We presumed that cells that were already polarized and migrating at the start of filming was due to fast cell polarization during the time it took to find cells and capture the first image. We excluded the rare cells that moved the wrong way from subsequent analyses. We were able to readily distinguish all these cell behaviors in careful analysis of movies. Thus, we readily identified polarizing cells that we captured in movies, and only analyzed those polarizing cells for our study (Table S1).

##### Limitations and Controls in the Assay

In order to answer the questions posed in the study we had to trade sufficient temporal resolution (typically 200-300 frames of movie at 1 frame/s) with shorter total length of movie (200-300 s = 3.3- 5 minutes). Once cells initiated cell migration and whole cell translocation, they continued migrating for the remaining period of the movie, sometimes with re-polarization and re-migration in the expected direction. We illustrate examples of continued monitoring of the same cells through initiation of cell migration and subsequent whole cell translocation in (Figures 1J and 1K compared with Figures S1G and S1H; and by inspection of images in Figures 1C–1E, 1H, 1I, 2C–2E, 3A, 3B, 4A, 4B, 4I, and S1D, and S1E). To compare behavior of AX2 cells with *mlcE* null cells we kept the period of filming the same and asked what proportion of cells initiated cell migration and subsequent whole cell translocation in that time period.

#### Cell Turning Assay in Live Cells

This assay reports cells induced to turn in a population of live migrating cells. We used cell turning as an alternate method to study breaking of symmetry. Aggregation competent *Dictyostelium* cells were prepared for filming as for the initiation of cell migration assay, except cells were not pre-cooled on ice, and to induce a cell turn the micropipette containing cAMP or 8CPT was moved to a new position that was filmed [19]. Images were collected every 1 s with DIC time-lapse microscopy using a Zeiss 710 laser scanning confocal microscope and a 63 × oil-immersion objective (Zeiss, Germany). We fully characterized and quantified the assay in (Table S2). Developmental stage was controlled the same for each cell turning assay. Further, for each cell that we tested, prior to moving the pipette, we ensured that that cell was fully polarized and undergoing whole cell translocation. Within this window of development and also dependent on how long each individual cell had been previously migrating for, migrating cell shape ranged from rounder to longer. We used cell turning to identify the temporal order of new cell front and new cell rear formation, therefore we only studied those cells that did turn and that had distinguishable cell boundaries and sequence of events (fully reported in Table S2).

### Quantification and Statistical Analysis

#### Source Films used for Cell Analyses

In most films in AX2 cells, cells were transformed with both actin and myosin II markers and dual fluorescence and DIC images acquired. Not all individual cells in all movies visibly expressed both markers, due to variable levels of expression cell to cell. For each experimental condition, we pooled polarizing cells (identified as described, above) for subsequent analysis. Then we separately analyzed cell margin displacement (Figures 1, 2, 3, and 4), actin filament localization (Figure 3), myosin II localization (Figure 4) and myosin II intensity (Figure 4). In further separate analysis, we analyzed actin and myosin II dynamics within the same cell in cells that sufficiently expressed both markers (Figure 4H).

#### Tracking Front and Rear Cell Margin Displacement

Live cells were analyzed one-by-one, manually in each movie, frame-by-frame in MetaMorph (Universal Imaging) [27, 46, 54, 55]. This is very labor intensive, but yields very accurate information on precise position of the cell margin, as required for the study. For all experiments, events were captured in live cells at a temporal resolution of 1 s that is significantly faster than the cell polarizes (roughly 30-60 s) thereby allowing the start of cell front protrusion to be readily distinguished from the start of cell rear retraction. We identified which end of the cell was the front and rear, and which of these margins displaced first, readily and unambiguously by tracking through the movie frame-by-frame and marking position of the cell margin with time, typically at 200%–300% magnification on screen.

Initial rear retraction manifest as either: retraction of a discrete, larger, cellular zone (for example, zone on the bulk cell body Figure 1H); or coordinate, or near coordinate retraction of several, smaller discrete zones, located near each other (e.g., delocalized protrusions in Figure 2D, black arrows); or both (e.g., Movie S4) in which case all discrete zones were tracked. Initial front protrusion tended to protrude from one contiguous (e.g., Movie S1) or near contiguous (e.g., Movie S2) cellular zone.

#### Morphometrics Displayed in the Figures

For manuscript space considerations, frames from the movies in the figures are illustrated at 1-4 s time intervals depending on either cell speed or duration of the delay between front and rear margin displacement. Distance-time graphs were plotted every 3 s as analysis showed that the delay between the front and rear was significantly longer than 3 s for these individual cells (Figures 1J and 1K) and an average 4–5 fold longer in the cell population (Figure 1G). Line scans of fluorescence intensity were acquired at a line width of 5 pixels from raw images at the indicated times in the figures and raw data displayed (Figures 3F and 3G). Kymographs were acquired at a line width of 3 pixels, every 1 s from raw time-lapse sequences (Figures 3C and 4C and 4D) and then scaled for illustration (below).

#### Scaling of Images in the Figures

All frames showing F-actin or myosin II fluorescence in cells and all frames that comprise kymographs, were scaled the same for any given individual cell and the same for all pixels in the image to allow fair comparison of fluorescence with spatial location and time for that cell. DIC images of cells were scaled to sufficiently increase the contrast so that the cell margins were clearly identifiable in reproduced images. For overlay images, scaled images were used for the source images. When comparing attractant and repellent, the parameters of the overlay were the same. Illustrated images in figures accurately reflect the original, raw images.

#### Fluorescence Intensity Measurements

Average integrated fluorescence intensity per unit area was measured in rectangles of approximate 3-6 μm^2^ and within paired rear and front zones in the same cell from raw fluorescence images. Figure 4A indicates the location of cell rear (bracket 1) and cell front (bracket 2) zones for measurements. The very tip of the cell front (∼1 μm; bracket 3) was excluded from measurements as myosin II is typically excluded from this zone in these and many other cells during whole cell translocation. The ratio of fluorescence intensities between relevant zones (recorded in Figures 4F and 4G) was then determined for each individual cell as that cell transited through key steps: non-polarized; initial displacement of the rear margin; whole cell translocation. The cell population average of individual ratios was then determined for each of these steps.

#### Location of Statistical Details

Details of all cell behaviors are located in Tables S1 and S2. Standard error of the mean and number of experiments are provided in the figures or results. n represents cells or cell margin zones as specified in the figure legends.

## Supplemental Information

Document S1. Figures S1 and S2 and Tables S1 and S2

Movie S1. AX2 Cell Polarizing and Migrating toward a Gradient of cAMP Attractant, Related to Figure 1 The cell is the one in Figure 1A (in the box) and C. cAMP is at the bottom of the screen, out of field of view (needle visible in Figure 1A). Initial cell front protrusion breaks cell symmetry and starts migration (front protrudes down the screen, beyond lower black line). Initial cell rear retraction at one cell side occurs next, then at the cell rear (rear retracts down the screen beyond the upper black line), both delayed relative to front protrusion. 15.6 s quick time movie = 156 s original elapsed time. Original images from live cells during the breaking of symmetry and initiation of migration were acquired at 1 frame every second. For presentation, quick time movies here were made from the acquired movie playing each frame for 1/10^th^ second. Digital contrast is the same in all frames for each movie.

Movie S2. AX2 Cell Polarizing and Migrating away from a Gradient of 8CPT Repellent, Related to Figure 1 The cell is the one in Figure 1B (in the box) and D. 8CPT is at the top of the screen, out of field of view (needle visible in Figure 1B). Initial cell rear retraction breaks cell symmetry and starts migration (rear retracts down the screen, beyond upper black lines). The start of front protrusion at the other cell-end is delayed relative to initial rear retraction (front protrudes down the screen beyond the lower black line). 10.6 s quick time movie = 106 s. Original elapsed time. original images from live cells during the breaking of symmetry and initiation of migration were acquired at 1 frame every second. For presentation, quick time movies here were made from the acquired movie playing each frame for 1/10^th^ second. Digital contrast is the same in all frames for each movie.

Movie S3. AX2 Cell Expressing the Actin Marker GFP-ABP-120 and Initiating Migration toward a Gradient of cAMP Attractant, Related to Figure 3 The cell is the one in Figure 3A, rotated roughly 90°. cAMP is at the bottom of the screen, out of field of view. An increase in F-actin at the prospective front cell margin (compare fluorescence with white line) links to front protrusion-initiated migration (front protrudes down the screen beyond white line) and rear retraction is delayed (rear retracts down the screen beyond black line). 4.0 s quick time movie = 40 s original elapsed time. Original elapsed time. original images from live cells during the breaking of symmetry and initiation of migration were acquired at 1 frame every second. For presentation, quick time movies here were made from the acquired movie playing each frame for 1/10^th^ second. Digital contrast is the same in all frames for each movie.

Movie S4. AX2 Cell Expressing the Actin Marker GFP-ABP-120 and Initiating Migration away from a Gradient of 8CPT Repellent, Related to Figure 3 The cell is the one in Figure 3B. 8CPT is left of screen, out of field of view. From 0 s of quick time movie: initial rear retraction starts cell polarization and migration (rear retracts rightwards beyond four short, black lines that mark zones on the rear at their position at 0 s). 0 s to ∼2.0 s of quick time movie: No detectable stable bias in F-actin at the opposite, prospective cell front occurs during initial rear retraction. 2.0-3 s of quick time movie: Stable, biased F-actin at the opposite, prospective cell front starts to visibly accumulate during the later phases of rear retraction. Also the prospective front is encroached upon from behind as the rear and organelle flow continues moving forward. F-actin has clearly polarized just after 3.0 s of quick time movie (appearance of long, vertical black line in the movie). From 3+s-4.5 s of quick time movie: delayed F-actin polarization at the prospective cell front (long, vertical black line) couples to delayed start of initial front protrusion (front protrudes rightwards, beyond vertical black line). 4.5 s quick time movie = 45 s original elapsed time. Original elapsed time. original images from live cells during the breaking of symmetry and initiation of migration were acquired at 1 frame every second. For presentation, quick time movies here were made from the acquired movie playing each frame for 1/10^th^ second. Digital contrast is the same in all frames for each movie.

Document S2. Article plus Supplemental Information
