# Supplementary material for: Repellent and Attractant Guidance Cues Initiate Cell Migration by Distinct Rear-Driven and Front-Driven Cytoskeletal Mechanisms
Source: Curr Biol. 2018 Mar 19;28(6):995–1004.e3. doi: 10.1016/j.cub.2018.02.024 (PMC5863766; doi:10.1016/j.cub.2018.02.024)
Supplement: Document S1. Figures S1 and S2 and Tables S1 and S2 [file mmc1.pdf]

Current Biology, Volume *28*

Supplemental Information

Repellent and Attractant Guidance Cues
Initiate Cell Migration by Distinct Rear-Driven
and Front-Driven Cytoskeletal Mechanisms

Louise P. Cramer, Robert R. Kay, and Evgeny Zatulovskiy

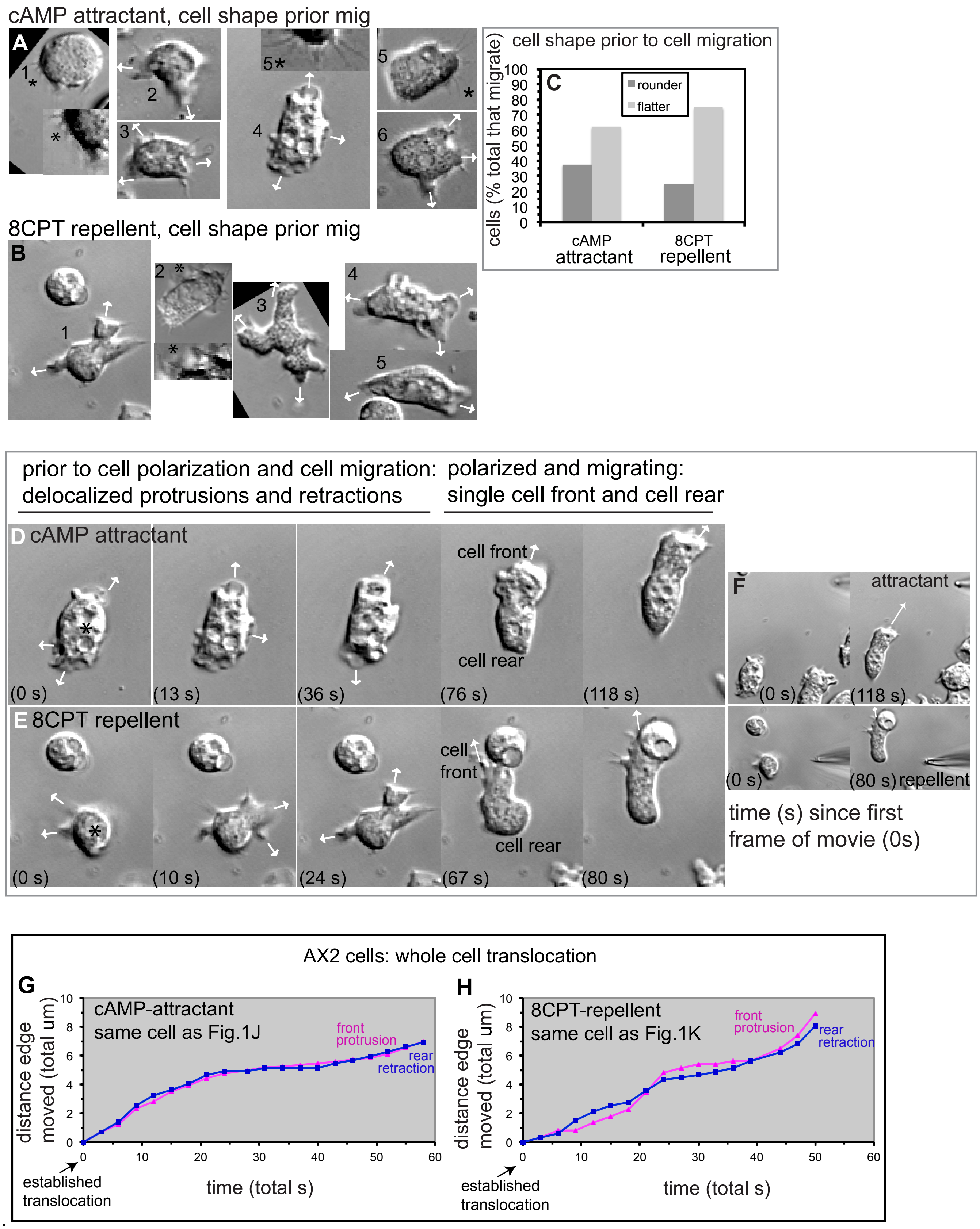

cAMP attractant, cell shape prior mig
A
8CPT repellent, cell shape prior mig
B
cell shape prior to cell migration
C
rounder
flatter
cells (% total that migrate)
cAMP attractant
8CPT repellent
prior to cell polarization and cell migration: delocalized protrusions and retractions
polarized and migrating: single cell front and cell rear
D cAMP attractant
cell front
cell rear
(0 s)
(13 s)
(36 s)
(76 s)
(118 s)
E 8CPT repellent
cell front
cell rear
(0 s)
(10 s)
(24 s)
(67 s)
(80 s)
F
attractant
(0 s)
(118 s)
(0 s)
(80 s) repellent
time (s) since first frame of movie (0s)
AX2 cells: whole cell translocation
G
cAMP-attractant
same cell as Fig.1J
front protrusion
rear retraction
distance edge moved (total um)
established translocation
time (total s)
H
8CPT-repellent
same cell as Fig.1K
front protrusion
rear retraction
distance edge moved (total um)
established translocation
time (total s)

I	**Wild-type (AX2)** cAMP *attractant*	**Wild-type (AX2)** 8CPT *repellent*
speed[a]	Protrusion:<br>12.50 +/- 0.92<br>Retraction:<br>12.10 +/- 0.91<br>(n = 14 cells)	Protrusion:<br>11.73 +/- 1.45<br>Retraction:<br>12.21 +/- 1.56<br>(n = 12 cells)
Ratio[b]	0.98 (n = 14)	1.02 (n = 12)

[a]Paired measurements of front protrusion and rear retraction in individual translocating cells (mean of individual cells μm/min +/-SEM). [b]average of ratios in individual cells.

Figure S1. Cell shape and behaviour before polarisation, after polarisation and during cell migration and related to Figure 1: AX2, live cells were cooled in the absence of cell guidance cue and then rewarmed in the presence of a gradient of cAMP attractant or of 8CPT repellent. Images are of cells in the presence of the guidance cue.

(A-H) Validation of protocol: cell shape and behaviour before and after polarisation.

(A, B) Two cell shape galleries, showing that similar to previous reports (cited in main text) cells in cAMP (A) and in 8CPT (B) adopt rounder and flatter non-polarised shapes. Numbers 1-3 (A) and 1-2 (B) indicate rounder shapes. Numbers 4-6 (A) and 3-5 (B) indicate flatter shapes.

(A, B) The cell shape galleries also show that random, delocalised, protrusions occur before cell polarisation: white arrows denote transient lamellipodia and pseudopodia and asterices, transient filopodia and microspikes. All cells shown subsequently polarise and migrate in the expected direction, irrespective of initial non-polarised shape or type of transient protrusion (see examples in D-E).

(C) Comparison of non-polarised shape of cells that subsequently migrate, 24 cells each guidance cue.

(D-E) Time-lapse sequences showing a cell transiting from non-polarised to polarised and migrating in response to cAMP (D) or 8CPT (E). Shown are the transient, delocalised protrusions and retractions that occur before polarisation (D, 0-36s; E, 0-24s; white arrows) and the subsequent, stable, polarised cell margin displacements at the front and rear that polarise cells (D, 76s; E, 67s; front and rear are labelled; white arrow indicates direction of migration). The main paper determines precisely which cell margin (front or rear) displaces first. This defines the break in cell symmetry (and the start of polarisation and migration). For reference, these cells began to polarise just after 36s (D, cAMP) and just after 24s (E, 8CPT) of filming. (F) shows needle position for (D, E). Time (s) is relative to the start of filming.

(G-I) Behaviour of opposite cell margins during whole cell translocation

(G, H) Distance-time plots: The same cells that were tracked during the start of polarisation and migration towards cAMP (in Figure 1J) and away from 8CPT (in Figure 1K) were then further tracked during subsequent whole cell translocation using the same time scales (G, cAMP) and (H, 8CPT) respectively. The plots show that unlike during cell polarisation (Figure 1) there is no obvious delay between front protrusion and rear retraction during whole cell translocation (G, H). 0 s (in G) corresponds to 55 s (in Figure 1J) and 0 s (in H) corresponds to 51 s (in Figure 1K).

(I) Table showing speed of front protrusion and rear retraction during whole cell translocation

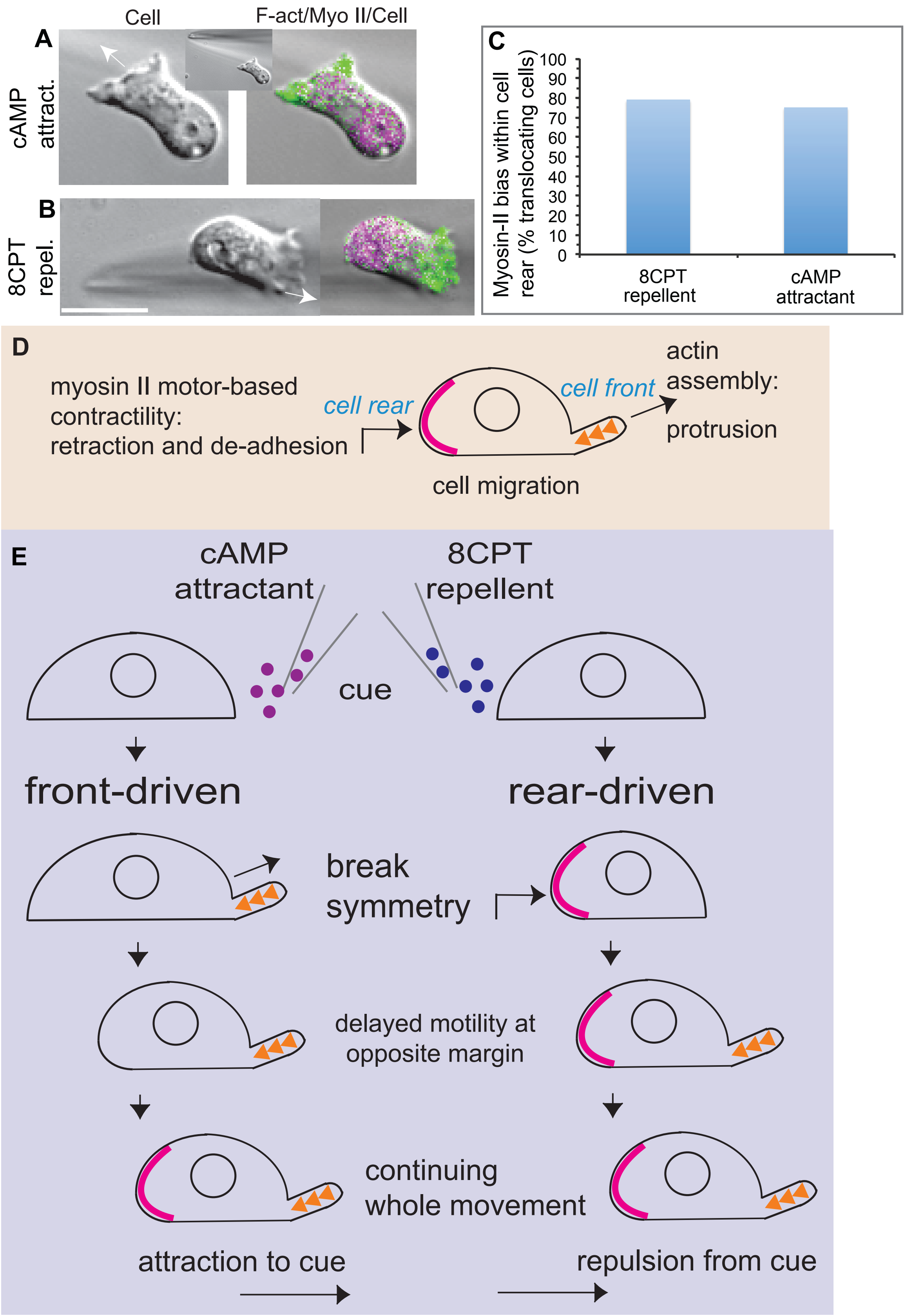

Cell
F-act/Myo II/Cell
A
cAMP attract.
B
8CPT repel.
C
Myosin-II bias within cell rear (% translocating cells)
100
90
80
70
60
50
40
30
20
10
0
8CPT repellent
cAMP attractant
D
myosin II motor-based contractility: retraction and de-adhesion
cell rear
cell front
actin assembly: protrusion
cell migration
E
cAMP attractant
8CPT repellent
cue
front-driven
rear-driven
break symmetry
delayed motility at opposite margin
continuing whole movement
attraction to cue
repulsion from cue

Figure S2. Distribution of F-actin and myosin II in AX2 amoeba in response to a gradient of guidance cue and related to Figures 1-4.

(A-C) Localisation of F-actin and myosin II fluorescence reporters in live AX2 cells during whole cell translocation and related to Figure 4**.**

(A, B) Images of an AX2 cell and triple overlay of paired cell, F-actin (green) and myosin II heavy chain (pink) fluorescence showing F-actin bias within the cell front and myosin II bias within the cell rear in AX2 cells moving towards cAMP (A) or away from 8CPT (B). The distribution of F-actin and myosin II is similar in both cases.

(C) Proportion of cells that have visible myosin II spatial bias within the rear in translocating cells (n= 24 cells, 8CPT; and 20 cells, cAMP).

(D-E) Diagram representing the data in the main paper during the breaking of cell symmetry

(D) Key of polarised cell margin displacement and cytoskeleton forces at the front and back of the cell for the images in (E).

(E) We discover front-driven and rear-driven modes of initiating migration in response to attractive and repulsive cell guidance cues. Local F-actin assembly drives front-driven breaking of cell symmetry towards cAMP-attractant, whereas local myosin II motor-based contractility drives rear-driven breaking of symmetry away from 8CPT-repellent. In the cAMP case, other contractility mechanisms (not shown) likely power the initial (delayed) rear formation.

Bar (B) = 10μm (A, B)

Number of Cells	cAMP attractant gradient	8CPT repellent gradient
Total assessed	87 (in 10 polarisation experiments)	141 (in 13 polarisation experiments)
Non-polarised at start of filming	73/87 (83.9%)	114/141 (80.9%)
Migrating at start of filming[a]	9/87 (10.3%)	20/141 (14.2%)
Other behaviours, total:[a]	5/87 (5.7%)	7/141 (5.0%)
Protrude axially	3	1
Collide or stick to another cell	1	2
Other	1, needle moved	4, multiple changes in behaviour
**Fate of cells, that are non-polarised at start of filming:**	Total = 73	Total = 114
Polarise and migrate towards attractant or away from repellent	24/69 (34.8%)	35/113 (31.0%)
Do not polarise[a]	45/69 (65.2%)	78/113 (69.0%)
Migrate wrong way[a]	4	1
**Precise mechanism, of those cells that polarise and migrate, towards attractant or away from repellent**	Total = 24	Total = 35
Cell front protrusion starts cell polarisation and migration	21/24 (87.5%)	3/35 (8.6%)
Cell rear retraction starts cell polarisation and migration	0/24 (0%)	26/35 (74.3%)
Cell front protrusion and cell rear retraction start together	3/24 (12.5%)	6/35 (17.2%)

[a] Precludes further assessment.

Table S1. **Behaviour of Each AX2 Cell in Initiation of Cell Polarisation Experiments: Filmed during first 200-300s Stable Encounter with Gradient at 1s Time Resolution and related to Figure 1.**

**Number of Cells**	**cAMP attractant gradient**	**8CPT repellent gradient**
Total assessed	86 (in 13 re-orientation experiments)	48 (in 18 re-orientation experiments)
Turn towards attractant or away from repellent	82/86 (95.3%)	41/48 (85.4%)
Do not turn[a]	4/86 (4.7%)	7/48 (14.6%)
**Of the cells that turn**	Total = 82	Total = 41
Turn with clear cell edge boundaries	71/82 (86.6%)	37/41 (90.2%)
Turn without identifiable cell edge boundaries or behaviour, total[a]:	11/82 (13.4%)	4/41 (9.8%)
	11/11 Turn in a group	3/4 movie cut short<br>1/4 complex behaviour
**Type of turn, of the cells that turn** with identifiable cell edge boundaries	Total = 71	Total = 37
Reverse or Lateral Turn (thus re-polarise to turn)	28/71 (39.4%)	36/37 (97.3%)
U-turn (thus do not re-polarise) [a]	43/71 (60.6%)	1/37 (2.7%)
**Precise mechanism of polarisation, of the cells that re-polarise to turn**	Total = 28	Total = 36
Cell front protrusion starts cell polarisation and migration	20/28 (71.4)	0/36 (0%)
Cell rear retraction starts cell polarisation and migration	2/28 (7.1)	28/36 (77.8%)
Cell front protrusion and cell rear retraction start together	6/28 (21.4%)	8/36 (22.2%)

[a] Precludes further assessment.

Table S2. **Behaviour of Each AX2 Cell in Cell Turning Experiments: Assessed at 1s Time Resolution and related to Figure 2.**
